# The Influence of Sociodemographic Factors on the Engagement of Citizens in the Detection of Dead Corvids During the Emergence of West Nile Virus in Ontario, Canada

**DOI:** 10.3389/fvets.2019.00483

**Published:** 2020-01-22

**Authors:** Andrea L. Thomas-Bachli, David L. Pearl, E. Jane Parmley, Olaf Berke

**Affiliations:** ^1^Department of Population Medicine, Ontario Veterinary College, University of Guelph, Guelph, ON, Canada; ^2^Canadian Wildlife Health Cooperative, University of Guelph, Department of Pathology, University of Guelph, Guelph, ON, Canada

**Keywords:** West Nile virus, surveillance, citizen science, arbovirus, wildlife disease surveillance

## Abstract

West Nile virus (WNv) was introduced into North America in 1999, and by 2002 was identified in most regions of Ontario, Canada. Surveillance of WNv included testing of corvids found dead and reported by citizens across Ontario, which at the time was a novel citizen science application for disease surveillance. While this surveillance program was successful for timely identification of WNv as it emerged and spread across the province, it is important to consider the influence of non-disease factors on surveillance data collected by the public. The objective of this study was to examine associations between rates of citizen phone reports of dead corvids and sociodemographic factors within the geographic areas where the reports were obtained. The data were grouped by forward sortation area (FSA), a geographical area based upon postal codes, which was linked with census data. Associations between the weekly rate of citizen reports and FSA-level sociodemographic factors were measured using multilevel negative binomial models. There were 12,295 phone call reports of dead corvids made by citizens in 83.3% of Ontario FSAs. Factors associated with the weekly rate of phone reports included the proportion of high-rise housing, the proportion of households with children, the proportion of seniors in the population, the proportion of citizens with no knowledge of either official language and the latitude of the FSA. There were higher rates of citizen phone reports in FSAs with <80% high-rise housing and greater proportions of households with children. A positive and negative association in the rate of calls with the proportion of seniors and latitude of the FSA, respectively, were moderated by the proportion of the population with knowledge of official language(s). Understanding the sociodemographic characteristics associated with citizen reporting rates of sentinels for disease surveillance can be used to inform advanced cluster detection methods such as applying the spatial scan test with normal distribution on residuals from a regression model to reduce confounding. In citizen-derived data collected for disease surveillance, this type of approach can be helpful to improve the interpretation of cluster detection results beyond what is expected.

## Introduction

West Nile virus was first identified in 1937 in Uganda (1), and until the mid-1990's was associated with sporadic outbreaks of mild illness in humans in Africa, the Middle East, and Europe (2). Subsequently, strains of WNv appeared to cause higher morbidity and mortality in humans and horses in parts of Europe, North Africa and the Middle East (3). A strain of WNv implicated in outbreaks of mortality in domestic geese in Israel was homologous to the strain that emerged in New York State, United States (U.S.) in 1999 (4). These outbreaks were associated with more severe neurologic disease and higher mortality in humans, horses and birds. Members of the corvid species, e.g., American Crows (*Corvus brachyrhynchos*), Black-billed Magpies (*Pica hudsonia*), Common Ravens (*Corvus corax)*, Blue Jays (*Cyanocitta cristata*), and Canada Jays (*Perisoreus canadensis)* were thought to be particularly susceptible to WNv infection, with American Crows, Black-billed Magpies and Blue Jays experimentally demonstrating high levels of viremia and mortality (5). In Canada, surveillance activities for the detection and monitoring of WNv were initiated in 2000, which included mosquito trapping and testing, sentinel surveillance using chickens, collection and testing of corvids found dead by the public, as well as reporting of human and equine cases (6). In addition, a database of all dead corvid phone reports in Ontario was created, which included the location of all corvid carcasses across Ontario, including those which were not tested for WNv and the test status of carcasses that had been collected (6). In 2001, the disease was first identified in Ontario in a found dead corvid, and subsequently, cases in corvids and humans were found across the province (and most of North America) through the summer and fall of 2002 (7).

Our previous research demonstrated that the citizen-derived data on dead corvids collected and tested provided timely identification of West Nile virus activity in public health units across Ontario, and demonstrated a high sensitivity for WNv detection (8). This was more evident during the early years after the incursion of WNv into Ontario, when public interest was high and naïve populations of corvids were highly susceptible to the disease. Furthermore, these data were useful for predicting where human cases would later occur, especially after adjusting for underlying sociodemographic and geographic factors associated with human cases (9).

There is increasing interest in citizen-derived data for scientific study (i.e., “citizen science”), including for the surveillance of wildlife diseases (10) and emerging vector-borne infections like Lyme disease (11) and Zika virus (12). Opportunistic citizen reporting can be a cost-effective option for data collection over wide geographic and temporal scales, including on private land (10). However, citizen-derived data can be biased by a number of factors including non-random distribution of effort and detection probability, which can influence their spatiotemporal distribution (13). The citizen phone reports of dead corvids during the initial emergence of WNv across Ontario in 2002 have not been explored for their associations with underlying area-level sociodemographic factors that may have influenced citizen participation in the detection and reporting of carcasses found in the environment. It is important to consider these potential confounding effects when advanced statistical methods such as cluster detection techniques are utilized for identification of higher-than-expected morbidity or mortality. While other potential explanatory factors such as other causes of mortality or varying underlying spatial distribution of corvids cannot be controlled, differences in the likelihood of citizens to report dead corvid carcasses when they are present may also bias these data if they are to be used for cluster detection. Specifically, adjusting cluster analyses for potentially confounding variables related to public participation can improve the identification of epidemiologically meaningful clusters. Understanding associations between citizen participation in data collection can also shed light on the potential level of citizen engagement among different population demographics during future disease emergence events, which can be used to improve communications soliciting citizen engagement and hence, their participation. Thus, the objective of this study was to examine the citizen phone reports of dead corvids in relation to area-level sociodemographic factors, in order to understand inherent biases in these particular data and to inform future research and communication strategies regarding the use of sentinel indicators for disease surveillance when participation by the public is requested.

## Methods

### Data Sources and Management

A dataset containing all citizen phone reports of found dead corvids across Ontario during 2002 was provided to the researchers by the Canadian Wildlife Health Cooperative (CWHC). This dataset included the date the dead corvid was found, whether the caller was a member of the public or from an organization, the street address, town/city and postal code, or geographic coordinates in latitude and longitude where the corvid carcass was located. The most consistently accurate level of spatial location for these entries at the highest geographic resolution available was the postal code information. In Canada, the first 3 characters of the postal code, i.e., the Forward Sortation Area (FSA), represents a geographic area within a major region or province/territory, based on mail distribution zones (14) and can be linked to Canadian Census data. Cartographic boundary files (14) and 2001 FSA Census data (14) were obtained from Statistics Canada. Phone reports which contained the latitude and longitude coordinates rather than street address and postal code were linked to the FSA within ArcMap v10.2.1 (ESRI, Redlands, CA, USA).

The latitude and longitude of the FSA centroid was determined using the Calculate Geometry function in ArcMap. Sociodemographic variables and human population size within the FSAs were obtained from the 2001 Canada Census (14). While the Census contains many potential explanatory variables, the sociodemographic variables chosen were based on the hypothesized non-overlapping biological or sociological influence on distribution of phone reports. The included the following variables: the proportion of high-rise homes (defined by Statistics Canada as a dwelling, owned or rented, in a building with 5 or more stories), the proportion of new homes (defined by Statistics Canada as those built between 1996 and early 2001 when the Census was collected), the proportion of households with children, and the proportion of low income households (defined by Statistics Canada as the percentage of economic families or unattached individuals who spend 20% or more of their income than average citizens on food, shelter, and clothing) (14). We also obtained the following Census sociodemographic variables concerning the proportion of the population that were: seniors (i.e., 65 years of age or older), in the labor force, had obtained a Bachelor's degree or higher education level, and had no knowledge of either official language (i.e., English and French) at the FSA level (14). Weekly phone reports of dead corvids within FSAs were calculated using Microsoft Excel (2016). These data were linked to the FSA centroid coordinates and FSA-level sociodemographic variables in a common dataset using Stata/SE version 14.0 (StataCorp, College Station, TX, USA).

### Statistical Analyses

#### Descriptive Statistics

The dead corvid reports were summarized by proportion of species reported (i.e., American Crows, Common Ravens, Blue Jays, Gray Jays (renamed Canada Jays), and non-corvid species). The phone call reports were summarized by number of calls per FSA, including those FSAs with no calls received over the study period.

#### Univariable Analyses

The dependent variable for this study was the FSA-level weekly phone call reporting rate, based on the number of weekly phone calls about dead corvids within each FSA. Because the outcome was rate-based and the data were over-dispersed, univariable negative binomial models were fit using Gaussian-Hermite quadrature (using the “menbreg” command in Stata). The models included an offset, which was the natural log of the number of people residing in the FSA, and a random effect with an independent covariance structure for FSA to control for clustering due to repeated observations within FSAs. Linearity was assessed using locally-weighted regression scatterplot smoothing (lowess) curves (15) between the FSA-level weekly rate of phone call reports and the following FSA-level continuous independent variables: FSA centroid latitude and longitude, the proportion of high-rise homes, the proportion of new homes, the proportion of households with children, and the proportion of low income households, as well as the proportions of the population that were seniors, in the labor force, had obtained a Bachelor's degree or higher education level, and had no knowledge of either official language. Any continuous variables found to have a non-linear relationship with these phone call reporting rates were categorized, if an appropriate transformation could not be found or the relationship could not be modeled with the addition of a quadratic term (15). Spearman's rank correlation coefficients (rs) were examined for each pair of independent variables to assess potential collinearity in subsequent multivariable modeling. If any pair of variables was found to be strongly correlated, using a cut-point of rs ≥ |0.8|, the variable considered most informative from a biological perspective was included in the proceeding models.

#### Multivariable Analyses

Multi-level negative binomial models were next fit by Gaussian-Hermite quadrature to explore the associations between the weekly citizen phone call reporting rates and FSA-level sociodemographic factors, in addition to the FSA centroid latitude and longitude. As for the univariable models, a random effect with an independent covariance structure for FSA was included to control for clustering due to repeated observations within FSAs and the natural log of the number of people residing in the FSA was the offset.

Based on the univariable models, variables that demonstrated an association with weekly citizen phone call reporting rates at a liberal significance level of alpha = 0.20 were further evaluated in multi-variable models using a backward step-wise elimination process. Associations and pair-wise interactions between variables considered for inclusion in the multi-variable model were assessed using two-tailed likelihood ratio (LR) tests at a significance level of alpha = 0.05. Assessment of confounding was performed by removing each non-intervening variable from the model and evaluating whether its removal resulted in a change in any coefficient by 20% or more. Coefficients were exponentiated and reported as incident rate ratios (IRR). Predicted curves were used to interpret interaction effects involving continuous variables. Fixed quantities were estimated for interaction terms on phone call reporting rates, while holding all other variables constant at their mean values.

Multi-variable model fit was evaluated by assessing the assumptions of normality and homogeneity of variance for the best linear unbiased predictors (BLUPs), graphically using a normal quantile plot and by a scatterplot of the BLUPs vs. the predicted outcome, respectively (15). Pearson and deviance residual plots were also examined to identify potential outliers (15). The final multi-level negative binomial model was also compared to a multi-level Poisson model and zero-inflated negative binomial model, using Akaike's Information Criterion (AIC) to identify the better fitting model for the data.

## Results

### Descriptive Statistics

There were 12,886 citizen phone call reports in Ontario concerning sightings of dead birds in 2002. The majority of bird species were classified as American Crows (95.64%), with the remainder classified as Blue Jays (0.99%), Common Ravens (0.53%), Gray Jays (0.02%), European Starlings (0.01%), Gulls (0.01%), and “other birds” (2.80%). There were 122 phone reports with either missing dates or location information (including postal codes), which were excluded from further analyses. Of the remaining records, the number of phone call reports about dead corvids within each FSA ranged from 0 to 407, with a mean of 21.8 and median of 6 calls. There were 85/510 (16.7%) FSAs where no citizen or organization reported dead corvids in 2002.

### Univariable Models

Based on univariable analyses, none of the variables were highly correlated with another variable. Those that were associated with phone call reporting rates based on a liberal *p*-value (*p* < 0.20) are displayed in Table 1. The following variables meeting these criteria were considered for inclusion in the multivariable model: FSA centroid latitude, the proportion of high-rise homes (categorized into two groups based on a cut-point of 80% high-rise), the proportion of new homes, the proportion of households with children, the proportion of the population with low income (modeled as a quadratic relationship), the proportion of seniors in an FSA, and the proportion of the population with no knowledge of either official language.

**Table 1:** Univariable^+^ associations between sociodemographic factors and rates of citizen phone call reports of dead corvids in Ontario during the West Nile virus outbreak of 2002.

**Sociodemographic variables**	**Incidence rate ratio (IRR)**	**95% confidence interval**	***P*-value**
Area with > = 80% high-rise dwellings Referent: area with <80% high-rise dwellings	0.37	0.27–0.50	<0.001
Proportion of households with children	1.06	1.05-1.07	<0.001
Proportion of seniors	1.14	1.11–1.16	<0.001
Proportion of citizens with no knowledge of either official language	0.92	0.86–0.98	0.013
Forward sortation area (FSA) centroid latitude	0.68	0.61–0.75	<0.001
Proportion of new homes	1.02	1.01–1.03	<0.001
Proportion of population with low-income	1.01^*^	0.95–1.07	0.76
Proportion of population with low-income squared	0.997^*^	0.996–0.999	0.004

*+Univariable mixed-effects negative binomial models. Forward sortation area (FSA) was included as a random intercept. Dependant variable, weekly number of phone call reports of dead corvids within the FSA. Offset, natural log-transformed number of people residing within the FSA. Only variables associated with phone call reporting rates based on a liberal p-value (p < 0.20) are displayed*.

* *Exponentiated coefficients for squared terms and their main effects rather than true IRRs*.

### Multivariable Models

**Figure 1:**
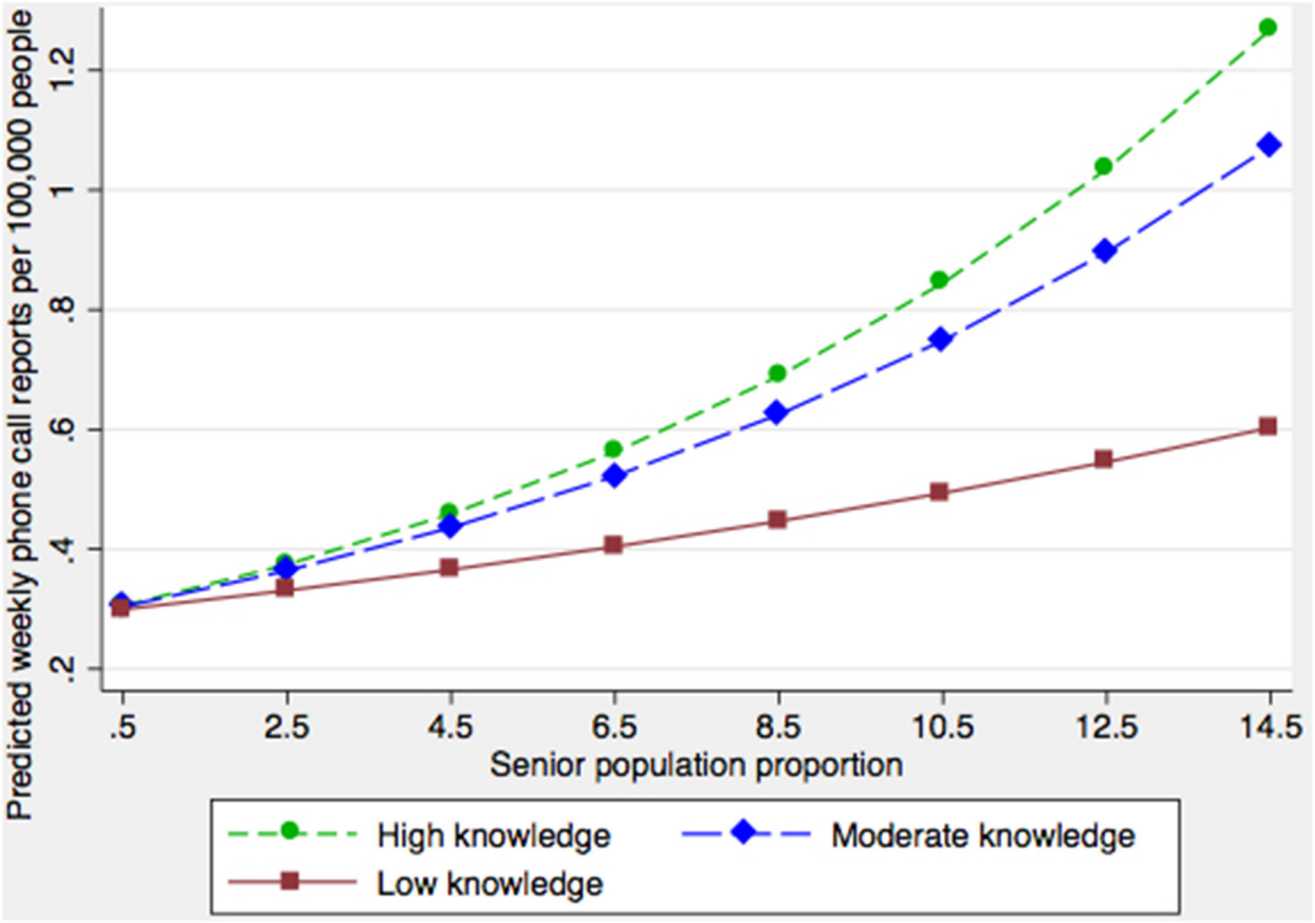
Model-adjusted predicted citizen call rates concerning dead corvids by forward sortation area (FSA) senior population proportion at three levels of official language knowledge. “High,” “Moderate” and “Low knowledge” classifications refer to the 25, 50, and 75th percentiles based on the proportion of the population within FSAs having no official language knowledge.

**Figure 2:**
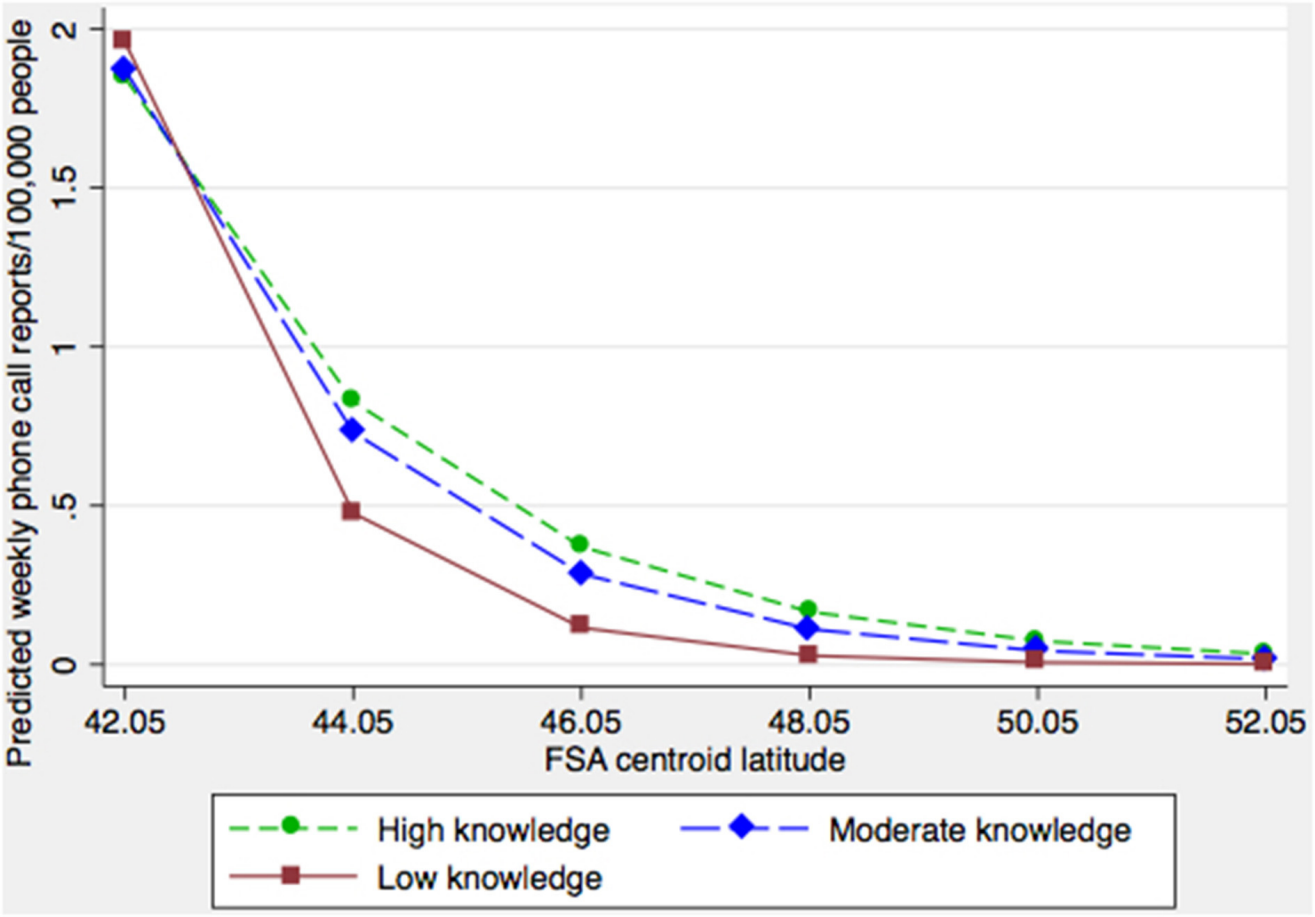
Model-adjusted predicted citizen call rates concerning dead corvids by FSA latitude at three levels of official language knowledge. “High,” “Moderate” and “Low knowledge” classifications refer to the 25, 50, and 75th percentiles based on the proportion of the population within FSAs having no official language knowledge.

Significant associations were identified (*p* < 0.05) between phone call reporting rates and the proportion of high-rises and the proportion of households with children (Table 2). There were interactions between the proportion of seniors and the proportion of people having no knowledge of either official language (Table 2), and the proportion of seniors and the latitude of the FSA (Table 2). The final fitted multi-level model demonstrated that, after controlling for other covariates, FSAs with >80% of households characterized as high-rise were associated with lower reporting rates (Table 2). Phone call reporting rates increased with the proportion of households with children (Table 2). Generally, as the proportion of seniors in the population increased, so did the rate of phone call reports about dead corvids. However, this increase was less rapid in FSAs where the proportion of the population speaking an official language was lower (Figure 1). For FSAs located at higher latitudes (i.e., further north), the predicted rate of phone call reports decreased, and there was a sharper decline in the rate of reports in FSAs where the proportion of people speaking either official language was lower (Figure 2).

**Table 2:** Multivariable^+^ associations between sociodemographic factors and rates of citizen phone calls reporting dead corvids in Ontario during the West Nile virus outbreak of 2002.

**Sociodemographic variables**	**Incidence rate ratio (IRR)**	**95% confidence interval**	***P*-value**
Area with > = 80% high-rise dwellings Referent: area with <80% high-rise dwellings	0.54	0.40–0.72	<0.001
Proportion of households with children	1.03	1.01–1.05	0.004
Proportion of seniors	1.09	1.05–1.13	<0.001
Proportion of citizens with no knowledge of either official language	3822.5^*^	16.73–873155.9	0.003
Forward sortation area (FSA) centroid latitude	0.74^*^	0.66–0.82	<0.001
Proportion of citizens with no knowledge of either official language × FSA centroid latitude	0.83^*^	0.73–0.94	0.003
Proportion of citizens with no knowledge of either official language × Proportion of seniors	0.97^*^	0.95–0.98	<0.001

*+Multi-variable mixed-effects negative binomial models. Forward sortation area (FSA) was included as a random intercept. Dependant variable, weekly number of citizen reports of dead corvids within the FSA. Offset, natural log-transformed number of people residing within the FSA*.

* *Exponentiated coefficients for interaction terms and their main effects rather than true IRRs*.

The best-fitting model based on AIC values was the negative binomial model with a random effect for the FSA. The evaluation of the residuals identified several outliers, but removal of these observations did not change the model estimates. The BLUPs for the random intercept for FSA were also normally distributed and demonstrated homogeneity of variance.

## Discussion

When West Nile virus appeared in North America, the reporting of dead corvids by citizens in various jurisdictions in North America provided a unique approach toward monitoring the spread of a vector-borne disease. This was made possible due to the high public concern about the implications of human West Nile virus infections, high mortality rates among corvid species infected, and their widespread distributions. The impetus for this study was to explore how human sociodemographic factors influenced rates of phone calls reporting corvids found dead by citizens in Ontario during 2002, when WNv first spread across the province.

We found that there was a high volume of citizen phone call reports of dead corvids received from most areas of Ontario during the spring, summer and fall of 2002. The majority of birds were classified by citizens as American Crow, which is consistent with previous studies on WNv-positive species distribution (16). Few birds were reported as non-corvid species, indicating the public was well-informed about which species to report, although most reports were not validated since the majority of specimens were not collected for testing (8). The citizen reports would have been influenced by a number of geographic and sociodemographic factors related to the likelihood of detecting corvid carcasses in the environment given their presence, and social factors related to an individual's knowledge of the WNv surveillance program requesting reports of dead crow carcasses and their willingness to report by phone any dead crows found. The number of households within FSAs may have also influenced these data if each phone call was placed by a single household, however, the population size of the FSA was used instead as an offset since phone call reports were placed by individuals, to control for the likely influence of FSA population size on number of phone call reports received. Household-level sociodemographic variables, available from the Census, were included to account for sociodemographic features of the small areas represented by FSAs.

Due to the ecological nature of this study, one must be cautious interpreting our findings from the community level (i.e., FSA) to the individual caller level. Small areas such as FSAs in Ontario, being more homogenous in their population structure can limit the effect of ecological bias (15). Furthermore, the latitude and longitude of the FSA were considered in relation to the weekly phone call reporting rates as a coarse measure of the geographic distribution of corvids, although we could not completely control for differential underlying distribution of corvids across the province, which would be influenced by the seasonal distribution patterns related to breeding, migration, and locations of roosting sites. Corvid distribution data are collected via other citizen participatory approaches, such as the North American Breeding Bird Survey (BBS) (17) and Project FeederWatch (18), but these point data are collected at a single timepoint along roadside survey routes (BBS) or during winter (Project FeederWatch), and were primarily developed for temporal trend analyses over a number of years. However, since corvids are widely distributed across the province and known to cohabit in proximity to humans (19), the following results suggest certain sociodemographic factors within small areas influenced the reporting of dead corvids by citizens in Ontario, given their presence in the surrounding environment.

We found that there were fewer phone call reports in areas with a high proportion of high-rises, in comparison to low-rise settings. This finding seems contrary to previous research which has shown that the American Crow (19) and *Culex* mosquitoes (20) are found in greater abundance in urban vs. rural areas. Also, research by Ward et al. (21), who used crow decoys to compare urban and rural differences in citizen detection and reporting rates and scavenging rates by other animals in a county in Georgia, U.S.A., found that detection and reporting was significantly higher in urban vs. rural areas, and carcass removal by scavengers occurred more quickly in rural parts of the country. It is intriguing then, that FSAs with a high proportion of high rises (as a surrogate measure for urbanicity) were associated with lower reporting rates in our study. Perhaps the cut-point of 80% high-rise density used in this study which was made based on the linear relationship with reporting rates, captured a different relationship in very high-density urban environments compared with all other regions. For example, areas with high density housing likely have fewer trees and may be further from woodlands for roosting, and this result may reflect a lower density of corvids. Lower abundance of mosquitoes and less transmission of WNv to corvids may provide another explanation, or anthropogenic factors related to the human population characteristics could explain the reduced likelihood of citizen detection and reporting of dead crows in these settings. For example, perceived safety and pleasurability of surrounding outdoor spaces has been found to be lower in high density neighborhoods, with a concurrent negative association with time spent outdoors (22).

We also found higher rates of phone call reports were associated with areas in which there were higher proportions of households with children. This finding may reflect the geographic features of areas with higher proportions of households with children, like suburban areas, being more suitable to corvid populations. It is also possible that citizens from households with children generally spend more time outdoors and are more engaged with public health messaging. Canadian children spend greater amounts of time outdoors relative to all other age groups (23), and other research has shown that children interact more directly with the natural environment in comparison with adults (24). Thus, this age group may be more likely to identify dead birds in their surrounding environment.

The association found between higher sightings rates and areas with higher proportions of seniors may reflect features of these FSAs promoting larger corvid populations since the majority of senior Canadians live in urban metropolitan areas (25). A national survey-based study does not support the presumption that Canadian seniors spend greater amounts of time outdoors in comparison with other Canadians (23), nor have they been found to participate more often than younger people in survey-based studies (26). Further research would be needed to better understand the underlying mechanisms behind higher reporting rates among FSAs with more senior residents.

The reduction in phone calls reports about dead corvids with increasingly northern, cooler latitudes likely reflects lower rates of WNv-infected crows due to mosquito-dependant WNv replication, amplification and transmission cycles. Further, FSAs in northern Ontario have generally lower human population densities, more open space, and likely more opportunities for scavenging of corvid carcasses by wildlife. Previous studies of anthropogenic causes of bird mortality have shown that the removal of bird carcasses (by scavengers) before an observer has a chance to detect them is one of the largest biases in estimating mortality rates using citizen-derived data (27, 28).

We also found that the associations between phone call reporting rates and the proportion of seniors in the population, as well as phone call reporting rates and the latitude of the FSA, varied by the level of knowledge of official languages within FSAs. It may be that in areas with higher proportions of new Canadians, there was lower awareness of West Nile virus risk and/or understanding of public health messaging requesting the reporting of found dead corvids. Other social and cultural barriers may have reduced the likelihood of citizens finding and/or calling to report dead corvids in areas where higher proportions of new Canadians reside. The finding that FSAs with higher proportions of seniors were associated with higher phone call reports is likely related to a number of reasons including having more available time and higher interest in, and knowledge of birds, although this effect was modified by the proportion of the population with knowledge of an official language.

It is likely that some citizens reporting dead corvids may have misclassified the species involved, since it may be difficult for average citizens to differentiate American crows, ravens and magpies from various other black species of birds (e.g., grackles and starlings). Geographic differences in bird species awareness among Ontario citizens may have produced unmeasurable biases in the data. In the current context, a mobile phone application could be developed to aid in identification of different species (29), and a photograph of the specimen could be uploaded by the user to verify the species and evaluate carcass quality for testing. There were also a high proportion of location entries with small errors that precluded the use of exact coordinates for analyses. Since we investigated associations at a small area level this was not a serious problem for the purpose of this study. Current widespread use of digital technologies, including smartphone mobile applications and GPS capabilities, would allow for relatively easy dead crow reporting, verifiable with respect to exact location and species identification. These technological advancements may improve reporting rates for future studies and surveillance programs and reduce some of the biases in citizen-derived wildlife data.

Citizen reporting of dead corvids in Ontario during the 2002 emergence and spread of WNv across the province depended on citizens having the knowledge and willingness to make phone reports, and on their likelihood of detecting the corvid species of interest if they were present. While the program was very successful at timely and sensitive identification of WNv in Ontario, the dead corvid reports collected from citizens, were non-random samples requiring careful handling in order to make valid spatial inferences about risk. Here, we identified sociodemographic factors related to the different rates of dead corvid reports among small areas in the province which may have been related to the likelihood of reporting, given the presence of the disease. Measures of WNv risk based on citizen reports of dead corvids should consider these potential factors influencing reporting, since statistical measures can be implemented to control for their confounding effects in epidemiological risk-based studies and cluster analyses (30, 31). Hence, it is important to consider underlying biases for any studies which utilize valuable citizen-derived data.

## Data Availability Statement

The datasets for this manuscript are not publicly available from the authors due to privacy restrictions. Requests for the caller data can be made to the CWHC. Census data are available from the Government of Canada.

## Ethics Statement

Ethical review and approval was not required for the study on human participants in accordance with the local legislation and institutional requirements. Written informed consent for participation was not required for this study in accordance with the national legislation and the institutional requirements. Ethical review and approval was not required for the animal study because Citizen-acquired phone calls about deceased corvids did not require ethical review and approval by the University of Guelph.

## Author Contributions

The study design and manuscript revision were completed by AT-B, DP, EP, and OB. Data analysis, interpretation and drafting of the manuscript were completed by AT-B.

### Conflict of Interest

The authors declare that the research was conducted in the absence of any commercial or financial relationships that could be construed as a potential conflict of interest.
